# Metformin therapy in patients with diabetes mellitus is associated with a reduced risk of vasculopathy and cardiovascular mortality after heart transplantation

**DOI:** 10.1186/s12933-019-0925-y

**Published:** 2019-09-16

**Authors:** Eilon Ram, Jacob Lavee, Alexander Tenenbaum, Robert Klempfner, Enrique Z. Fisman, Elad Maor, Tal Ovdat, Sergei Amunts, Leonid Sternik, Yael Peled

**Affiliations:** 1 Leviev Cardiothoracic and Vascular Center, Sheba Medical Center, Tel Hashomer, 52621 Ramat Gan, Israel; 2 Sackler School of Medicine, Tel Aviv University, Tel Aviv, Israel; 3 Israeli Association for Cardiovascular Trials, Ramat Gan, Israel

**Keywords:** Heart transplantation, Metformin, Cardiac allograft vasculopathy, Cardiovascular mortality

## Abstract

**Background:**

Cardiac allograft vasculopathy (CAV) is a major cause of morbidity and mortality following heart transplantation (HT). Reduced cardiovascular mortality and morbidity have been reported in non-HT patients treated with metformin. Given the high prevalence of type 2 diabetes mellitus (T2DM) in HT patients, we investigated the association between metformin therapy and cardiovascular outcomes after HT.

**Methods:**

The study population comprised 103 DM patients who had undergone HT between 1994 and 2018 and were prospectively followed-up. We excluded from the study patients with type 1 diabetes mellitus. Fifty-five HT patients (53%) in the cohort were treated with metformin. Clinical data were recorded on prospectively designed forms. The primary outcomes included CAV, survival, and the combined end-point of CAV or cardiovascular mortality.

**Results:**

Kaplan–Meier survival analysis showed that the CAV rate at 20 years of follow-up was lower in DM patients treated with metformin than in those who were not (30 vs. 65%; log-rank p = 0.044). Similarly, the combined risk of CAV or cardiovascular mortality was lower in the metformin-treated patients than in those not receiving metformin (32 vs. 68%; log rank p = 0.01). Consistently, multivariate analysis adjusted for age and comorbidities showed that metformin therapy was independently associated with a significant 90% reduction (95% confidence interval 0.02–0.46, p = 0.003) in the risk for the development of CAV, and a 91% reduction (95% confidence interval 0.02–0.42; p = 0.003) in the risk for CAV or cardiovascular mortality.

**Conclusions:**

In diabetic HT patients, metformin therapy is independently associated with a significant reduction in the long-term risk for CAV and the combined end-point of CAV or cardiovascular mortality after HT.

## Background

Cardiac allograft vasculopathy (CAV) is a major cause of morbidity and mortality after heart transplantation (HT) and remains a major obstacle to the long-term success of HT. The International Society of Heart and Lung Transplantation (ISHLT) registry reports a high incidence of CAV after HT: up to 50% by 10 years post-transplant and ~ 30% by 5 years post-transplant [1, 2]. Perhaps the most important information to emerge from an analysis of the ISHLT statistics is that in the past two decades there has been only a minimal reduction in 5-year CAV—from 32 to 29% [1, 2]. CAV is a diffuse panarteritis with concentric, longitudinal intimal thickening of the epicardial coronary arteries. It likely involves the coronary microvasculature as well. While CAV is generally a diffuse process, it can manifest in ways similar to native coronary artery disease with focal stenosis. The pathogenesis is multifactorial and involves contributions from atherosclerotic mechanisms, ischemia–reperfusion injury, immune responses, and particular infections. Although improvements in both immunological and nonimmunological interventions have been achieved in the field of HT, they have barely impacted the natural history of CAV. As such, the 5-year survival for patients with CAV detected within 3 years of transplant has improved marginally from to 71 to 76% but remains lower than the 82% survival for recipients without CAV [2].

A common post-HT complication—and a major contributor to morbidity and mortality following HT—is the development of type 2 diabetes mellitus (T2DM), with 21% and 35% of survivors being affected within 1- and 5 years following HT, respectively. These patients are usually treated with metformin, one of the most commonly used anti-diabetes drugs worldwide and generally the initial oral agent of choice for patients with T2DM. The potential benefits of this drug include: anti-glycemic efficacy, potential for weight reduction, attenuation of metabolic syndrome, lipid lowering benefits, anti-inflammatory effects both alone [3] or in combination with other medications [4] and anti-neoplastic potential [5, 6]. It also could limit the expansion of abdominal aortic aneurysms [7]. Increasing evidence is also accumulating that metformin has potential as a treatment for cardiovascular disease, since lower cardiovascular-associated mortality and morbidity have been reported in non-HT patients treated with metformin [8–12]. Thus, given the high prevalence of CAV and diabetes in HT patients and the favorable metabolic profile of metformin, we designed a study to investigate the association between metformin therapy and the incidence of CAV after HT.

## Methods

### Study design and participants

We conducted a retrospective cohort study of all patients over 18 years of age who had undergone primary HT and follow-up at our Center between 1994 and 2018. We excluded from the study patients who died within the first 3 months post-transplant, patients with type 1 diabetes mellitus, and patients treated with metformin following the diagnosis of CAV. Clinical data were recorded on prospectively designed forms and included comprehensive information regarding the transplantation procedure, immunosuppression protocol, occurrence of major cardiac events, malignancy, diabetes, and all treatments during long-term follow-up.

The institutional protocol for immunosuppression was consistent during the time period covered by the study. All transplant recipients received anti-thymocyte globulin induction therapy, followed by a triple-drug regimen comprising steroids, an antimetabolite, and a calcineurin inhibitor. In a small number of patients, conversion to a low dose of calcineurin inhibitor combined with everolimus was instituted. The considerations for conversion to everolimus were dictated by the patient’s risk profile, including cytomegalovirus infection, renal failure, allograft vasculopathy and malignancy risk.

T2DM was defined on the basis of the American Diabetes Association diagnostic criteria that were current at the time of diagnosis: hemoglobin A1c level of ≥ 6.5%; fasting plasma glucose level of ≥ 126 mg/dl; or random plasma glucose level of ≥ 200 mg/dl. Diabetes was managed in accordance with conventional treatment recommendations, which included lifestyle modifications, weight control, increased physical activity, diabetes education, and pharmacologic therapy prescribed in accordance with the appropriate recommendations [5, 13]. In this context, we note that despite significant changes in treatment guidelines for diabetes over the past 25 years, metformin has remained the drug of choice for the management of T2DM. Of importance, metformin was approved and available in Israel throughout the study period.

In the present study, patients with T2DM were divided into two groups according to whether or not they were treated with metformin. There were no contraindications (metabolic acidosis, severe renal or hepatic impairment and/or advanced congestive heart failure) for metformin treatment in any of the T2DM patients included in the study. The study was approved by our institutional review board.

### Outcomes

The primary outcomes for this analysis were freedom from CAV and survival. The secondary outcome was combined end-point CAV or cardiovascular mortality. CAV was diagnosed by coronary angiography and invasive hemodynamic assessment performed annually, along with clinical assessment and echocardiography, according to the recommended nomenclature for CAV of the ISHLT consensus statement [14]. Mortality data were obtained from the Population Registry of the State of Israel, where all deaths are required by law to be registered.

### Statistical analysis

Data are presented as mean ± standard deviation if normally distributed, or as median values and interquartile ranges. Continuous variables were tested by the Kolmogorov–Smirnov test for normal distribution. Categorical variables are expressed as frequencies and percentages. The groups were tested with the χ^2^ test for categorical variables and with a t-test or Mann–Whitney–Wilcoxon test, as appropriate, for normal/non-normal distributed continuous variables.

CAV and mortality outcome curves, by metformin therapy, were constructed according to the Kaplan–Meier method, and the curves were compared using the log-rank test. In these analyses, time to event follow-up started at the date of initiation of therapy with metformin or of the development of T2DM (for T2DM patients not treated with the drug).

To explore the independent association of metformin treatment and outcomes, we used the multivariate Cox proportional hazards model with time-dependent covariates. In the multivariate analysis, we included: (1) covariates that differed significantly in a univariate analysis; and (2) covariates that were clinically relevant to the outcomes. Therefore, the Cox proportional hazards models included the following covariates: metformin treatment, recipient age, etiology of the heart failure, hypertension, history of smoking, dyslipidemia, donor age, clinical cytomegalovirus disease, number of rejections grade 2 or higher, and gender. Statistical analyses were conducted using R foundation (version 3.5.1) [15].

## Results

### Study cohort

Of the original population of 298 patients who underwent HT, 39 patients who died within the first 3 months and 10 children under the age of 16 years were excluded from the analysis. Of the remaining patients, 146 (58.6%) patients did not have T2DM and were not treated with metformin. Of the remaining 103 diabetic patients (mean age 53 ± 9 years) that constituted our study population, 55 were treated with metformin and 48 were not. Of the 103 T2DM patients, 49 (48%) had the condition before HT, and 54 (52%) developed T2DM after HT. Baseline clinical characteristics of the patients in the two groups are presented in Table 1. Baseline patient and donor clinical and demographic characteristics were similar for the two groups, except for a higher frequency of pre-HT T2DM in the metformin group.

**Table 1:** Baseline characteristics of the cohort

	Non-metformin N = 48	Metformin N = 55	p-value
Recipient age (years) (mean ± SD)	53 ± 11	54 ± 9	0.503
Donor age (years) (mean ± SD)	35 ± 13	33 ± 12	0.715
Recipient gender (male) (%)	42 (87)	47 (85)	0.989
Donor gender (male) (%)	23 (66)	29 (71)	0.825
Etiology (ischemic) (%)	38 (81)	35 (64)	0.089
Recipient BMI (kg/m^2^) (mean ± SD)	31 ± 33	27 ± 4	0.365
Donor BMI (kg/m^2^) (mean ± SD)	26 ± 5	27 ± 5	0.263
Hypertension (%)	27 (56)	36 (65)	0.451
Dyslipidemia (%)	28 (60)	43 (78)	0.069
Past smoker (%)	24 (50)	31 (56)	0.654
Assist device (%)	5 (10)	11 (20)	0.268
Status 1 (%)	26 (54)	29 (53)	1.000
PRA > 30% (%)	0 (0)	1 (2)	1.000
Recipient blood type (%)			0.664
A	14 (41)	12 (32)	
AB	5 (15)	4 (10)	
B	5 (15)	9 (24)	
O	10 (29)	13 (34)	
Recipient creatinine	1.5 ± 1.4	1.4 ± 0.6	0.524
Recipient bilirubin	1.7 ± 3.4	1.2 ± 0.9	0.317
Immunosuppression (%)			0.053
1	38 (79)	31 (57)	
2	10 (21)	22 (41)	
3	0 (0)	1 (2)	
Ischemic time (min) (mean ± SD)	149 ± 39	165 ± 39	0.118
PAM (mmHg) (mean ± SD)	29 ± 12	27 ± 13	0.382
CO (mean ± SD)	3.6 ± 1	3.6 ± 1.1	0.972
PVR (mean ± SD)	3.1 ± 1.8	2.7 ± 1.6	0.394
CMV mismatch (%)	8 (29)	7 (26)	1.000
Statins post-HT (%)	46 (96)	53 (96)	1.000
Hypertension post-HT (%)	47 (98)	51 (93)	0.446

*SD* standard deviation, *BMI* body mass index, *PRA* panel reactive antibody, *PAM* mean pulmonary pressure, *CO* cardiac output, *PVR* pulmonary vascular resistance, *CMV* cytomegalovirus, *HT* heart transplantation

### Risk for CAV

**Fig. 1:**
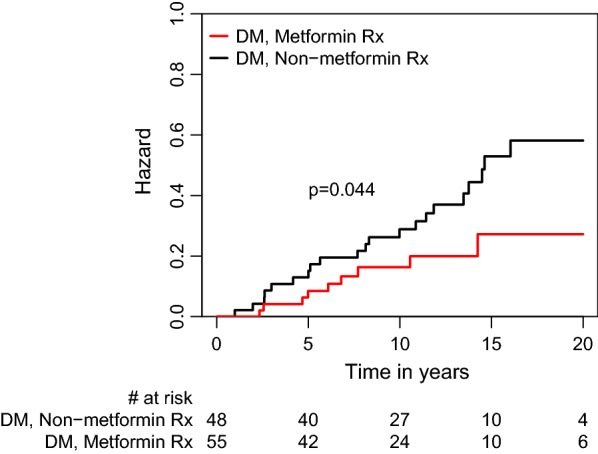
Kaplan Meier curves for 20-year freedom from cardiac allograft vasculopathy in recipients who did and did not receive metformin. *DM* diabetes mellitus

**Fig. 2:**
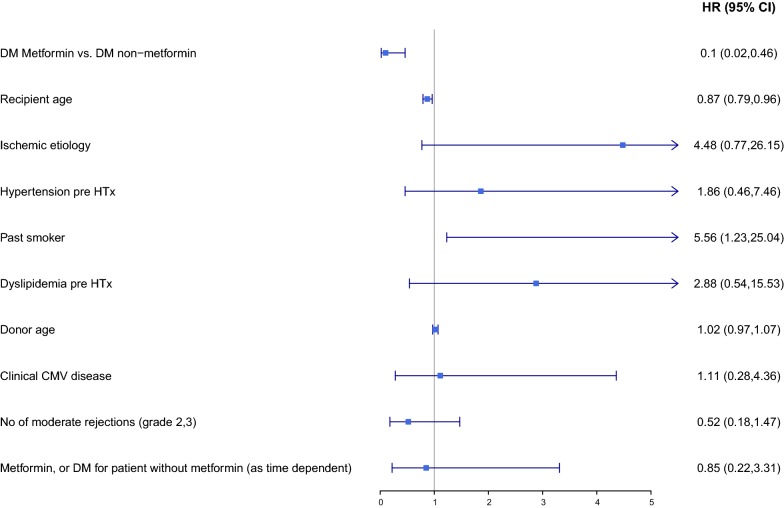
Forest plot of Cox regression: multivariate analysis-predictors for CAV. *CAV* cardiac allograft vasculopathy, *HR* hazard ratio, *CI* confidence interval, *DM* diabetes mellitus, *HTx* heart transplantation, *CMV* cytomegalovirus

Kaplan–Meier survival analysis showed that at 20 years of follow-up CAV-free survival was significantly higher in the metformin group than in the non-metformin group (60 vs. 35%, log-rank *p* = 0.044; Fig. 1). Multivariable analyses adjusted for age and comorbidities, using metformin as a time-dependent covariate, consistently demonstrated that metformin therapy was independently associated with a significant 90% reduction (95% confidence interval [CI] 0.02–0.46, p = 0.003) in the risk for the development of CAV (Fig. 2).

### Risk for combined end-point CAV or cardiovascular mortality

**Fig. 3:**
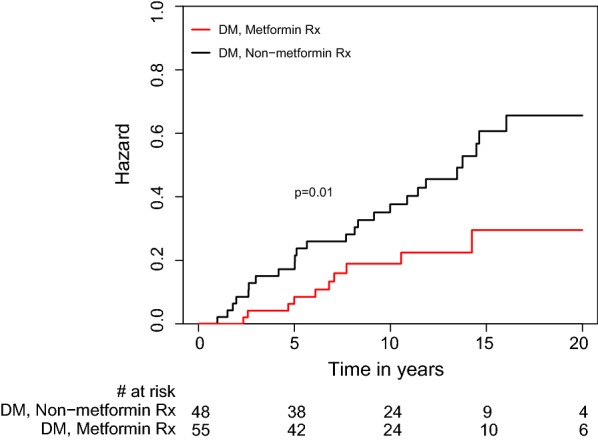
Kaplan Meier curves for 20-year freedom from composite cardiac allograft vasculopathy or cardiovascular mortality in recipients who did and did not receive metformin. *DM* diabetes mellitus

**Fig. 4:**
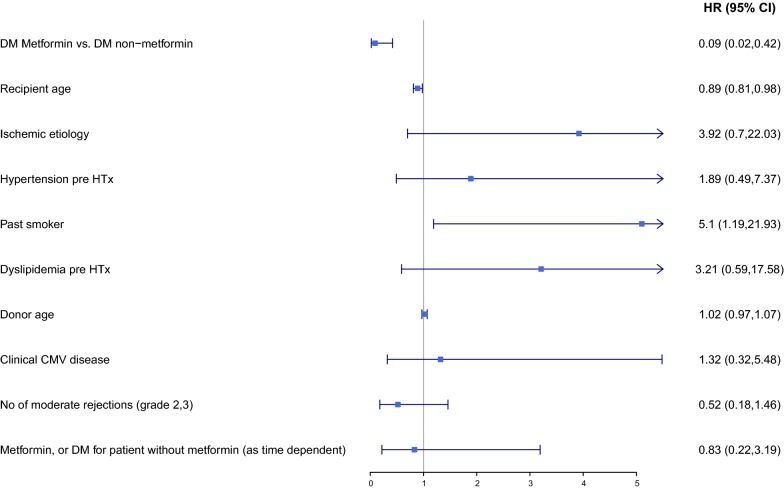
Forest plot of Cox regression: multivariate analysis-predictors of combined end-point of cardiac allograft vasculopathy or cardiovascular mortality. *CAV* cardiac allograft vasculopathy, *HR* hazard ratio, *CI* confidence interval, *DM* diabetes mellitus, *HTx* heart transplantation, *CMV* cytomegalovirus

Kaplan–Meier estimates of combined end-point of CAV or cardiovascular mortality are shown in Fig. 3. The combined risk for CAV or cardiovascular mortality was lower in the metformin-treated patients (32% vs. 68%; log rank p = 0.01). Consistently, multivariate analysis adjusted for age and comorbidities, using metformin as a time-dependent covariate, showed that metformin therapy was independently associated a 91% reduction (95% CI 0.02–0.42; p = 0.003) in the risk for CAV or cardiovascular mortality (Fig. 4).

## Discussion

The results of this investigation, designed to elucidate the influence of metformin on CAV, indicate that metformin therapy is independently associated with a reduced risk for CAV and combined endpoint of CAV or cardiovascular mortality. The importance of this study lies in the notion that CAV and diabetes are major confounders of mortality and morbidity after HT and therefore every effort should be made to reduce their burden. Thus, our findings could have major clinical implications for the treatment of HT patients, considering metformin treatment in patients with and without T2DM.

Although many strategies have been implemented to reduce CAV in HT recipients, in the past two decades there has not been any significant improvement in survival beyond 1 year, probably because the challenges in detecting and treating the processes underlying mortality, particularly those relevant to CAV, remain to be resolved [16]. It is currently held that the breakthroughs needed for CAV treatment will be derived from the growing understanding that CAV is initiated and propagated by both immunological and nonimmunological factors. With regard to the former, it is known that the traditional cardiovascular risk factors contribute to atherogenesis through enhancement of endothelial inflammation, leading to endothelial injury and fibroproliferative cellular responses [17]. Nonimmunological insults predisposing to CAV include vascular risk factors, and prominent among them is T2DM, frequently encountered in the post-HT course, with 21% and 35% of survivors being affected within 1 and 5 years following HT, respectively [18]. For the total cohort, approximately 40% of recipients were diagnosed with T2DM through the follow up.

Post-transplant diabetes is usually managed in accordance with the general guidelines for the treatment of T2DM in the general population [19, 20]. Metformin, the first-line oral agent used to treat patients with T2DM in the nontransplant population, has been shown to be safe for use in renal and cardiac transplant recipients [5]. By virtue of its potential non-hypoglycemic benefits, this therapy also appears to be the drug of choice for the HT population. These potential benefits include: attenuation of metabolic syndrome, cardiovascular protection, lipid-lowering benefits, neutral weight maintenance or potential weight reduction, and anti-neoplastic potential [5, 6, 21]. Furthermore, metformin is not metabolized by CYP3A4, and therefore there are no drug–drug interactions with immunosuppressive medications.

Various lines of evidence suggest that metformin has potential as a treatment for cardiovascular disease in both T2DM and non-T2DM patients. A landmark study of the cardiovascular benefits of metformin—the United Kingdom Prospective Diabetes Study (UKPDS) [22]—demonstrated that metformin reduces diabetes-associated deaths and all-cause mortality vs. any other conventional treatment [23]. Moreover, it shows synergistic effects with saxagliptin helping to improve endothelial dysfunction in early diabetes before macrovascular complications appear [24], protects the heart against hypertrophic and apoptotic remodeling after myocardial infarction [25] and is independently associated with a lower below-the-knee arterial calcification score [26]. Observational studies have also reported cardiovascular benefits in metformin users, especially in patients with T2DM and heart failure [27, 28].

Indications of cardiovascular benefits in metformin-treated T2DM patients have thus driven interest in repurposing metformin to treat cardiovascular disease, irrespective of diabetes status [29]. In non-diabetes patients who have cardiac syndrome X with normal coronary arteries but two consecutive positive exercise tolerance tests, an 8-week period of metformin treatment improved maximal ST-segment depression, Duke score, and chest pain incidence in comparison with placebo [9]. In the recently published prospective randomized control MET-REMODEL trial, metformin treatment of non-T2DM patients with coronary artery disease significantly reduced left ventricular mass index, left ventricular mass, systolic blood pressure, body weight and oxidative stress. As left ventricular hypertrophy is a good surrogate marker for cardiovascular outcome, that study did indeed indicate a cardioprotective role for metformin [30]. In addition, metformin usage was independently associated with lower coronary artery calcification scores in T2DM patients [31]. To the best of our knowledge, the current study is the first to investigate the effect of metformin on CAV in HT recipients.

The details of metformin’s cellular mechanism are yet to be elucidated definitively [29], but it is known that its fundamental mode of action is to reduce mitochondrial oxidative phosphorylation, thereby inducing energy stress [32] and inhibition of mitochondrial enzymes. While no detectable differential microRNA expression in non-atherosclerotic arteries of T2DM patients treated or untreated with metformin was found [33], it is known that mitochondrial suppression of oxidative phosphorylation activates AMP-activated protein kinase, selectively targeting redox control [34]. Importantly, it is also possible that the benefits of the drug in cardiovascular disease may be due to mechanisms distinct from its metabolic activity [29]. Metformin’s anti-inflammatory effects derive from its suppression of the nuclear factor κB (NF-κB) inflammatory signaling pathway [10, 35] and systemic inflammation markers. Metformin has also been found to suppress plasma cytokines—including the aging-associated cytokine, C–C motif chemokine ligand 11 (CCL11)—in patients with heart failure who do not have T2DM. It is known that blockade of CCL11 can suppress certain aspects of age-related cellular dysfunction.

The management of CAV currently remains limited and incomplete [36]. It is, however, well known that traditional atherosclerotic risk factors lead to CAV progression and, as such, the management of glycemia is thus of the utmost importance. Indeed, in keeping with this idea, we demonstrated that metformin therapy is independently associated with a significant reduction in the long-term risk for CAV and the combined end-point of CAV or cardiovascular mortality after HT.

There are several limitations to our study. First, there is the inherent limitation of observational trials that uncover associations but preclude the determination of cause-and-effect relationships. Second, our current practice does not include routine intravascular ultrasound assessment, which might be associated with underestimation of CAV. Third, this study was limited by being based on a single-center experience. Finally, the study sample was relatively small and not all possible confounders might have been recorded or taken into account. The present results will therefore require confirmation in larger cohorts and preferably with a prospective study design.

## Conclusion

Based on metformin’s cardiac and metabolic benefits, consideration should be given to the clinical implications of metformin therapy for HT recipients, even those without T2DM (unless there are contraindications), with focus on the reduction of CAV—a major obstacle to the long-term success of HT. The role of metformin therapy for reduction of cardiovascular morbidity and mortality in HT patients must be further evaluated prospectively.

## Data Availability

Data collected from a departmental database.

## Abbreviations

CAV: cardiac allograft vasculopathy
HT: heart transplantation
ISHLT: International Society of Heart and Lung Transplantation
T2DM: type 2 diabetes mellitus
CCL11: C–C motif chemokine ligand 11
